# Recent Emergence of Bovine Coronavirus Variants with Mutations in the Hemagglutinin-Esterase Receptor Binding Domain in U.S. Cattle

**DOI:** 10.3390/v14102125

**Published:** 2022-09-27

**Authors:** Aspen M. Workman, Tara G. McDaneld, Gregory P. Harhay, Subha Das, John Dustin Loy, Benjamin M. Hause

**Affiliations:** 1 United States Department of Agriculture (USDA) Agricultural Research Service (ARS), US Meat Animal Research Center (USMARC), State Spur 18D, Clay Center, NE 68933, USA; 2 Veterinary & Biomedical Sciences, South Dakota State University, Brookings, SD 57007, USA; 3 Nebraska Veterinary Diagnostic Center, School of Veterinary Medicine and Biomedical Sciences, University of Nebraska-Lincoln, 4040 East Campus Loop N, Lincoln, NE 68503, USA

**Keywords:** bovine coronavirus, hemagglutinin-esterase, variant, bovine respiratory disease, calf diarrhea

## Abstract

Bovine coronavirus (BCoV) has spilled over to many species, including humans, where the host range variant coronavirus OC43 is endemic. The balance of the opposing activities of the surface spike (S) and hemagglutinin-esterase (HE) glycoproteins controls BCoV avidity, which is critical for interspecies transmission and host adaptation. Here, 78 genomes were sequenced directly from clinical samples collected between 2013 and 2022 from cattle in 12 states, primarily in the Midwestern U.S. Relatively little genetic diversity was observed, with genomes having >98% nucleotide identity. Eleven isolates collected between 2020 and 2022 from four states (Nebraska, Colorado, California, and Wisconsin) contained a 12 nucleotide insertion in the receptor-binding domain (RBD) of the HE gene similar to one recently reported in China, and a single genome from Nebraska collected in 2020 contained a novel 12 nucleotide deletion in the HE gene RBD. Isogenic HE proteins containing either the insertion or deletion in the HE RBD maintained esterase activity and could bind bovine submaxillary mucin, a substrate enriched in the receptor 9-*O*-acetylated-sialic acid, despite modeling that predicted structural changes in the HE R3 loop critical for receptor binding. The emergence of BCoV with structural variants in the RBD raises the possibility of further interspecies transmission.

## 1. Introduction

Bovine coronavirus (BCoV; family *Coronaviridae*, genus *Betacoronavirus,* subgenus *Embecovirus* lineage A, species *Betacoronavirus 1*) is an economically important pathogen that causes enteric and respiratory infections in cattle worldwide. Mebus and colleagues first described BCoV in the early 1970s as a cause of neonatal calf diarrhea [1]. BCoV is now recognized to be involved in the etiology of at least three distinct clinical syndromes: enteric disease with high mortality in neonatal calves, winter dysentery with hemorrhagic diarrhea in adult cattle, and respiratory tract infections in cattle of all ages (reviewed in [2]). Respiratory infections also contribute to the development of the polymicrobial disease known as bovine respiratory disease complex (BRDC), which is one of the most economically important infectious diseases in cattle, and costs the U.S. alone more than a billion dollars annually [3]. Neonatal calf diarrhea is also of great economic significance and is estimated to be responsible for more than half of the calf mortality in dairies [4]. 

The BCoV genome is a single-stranded, non-segmented, positive-sense RNA of approximately 31 kb [5]. The 5′ two-thirds of the genome consists of two large overlapping open reading frames (ORFs), ORF1a and ORF1b, which encode the nonstructural replicase polyproteins (pp) 1a and pp1ab. Expression of pp1ab occurs due to a short ‘slippery’ sequence (UUUAAAC) in the RNA followed by a pseudoknot structure that is believed to direct a −1 RNA-mediated ribosomal frameshift during translation [6]. These two polyproteins are then proteolytically processed by virus-encoded proteases into several non-structural proteins involved in RNA synthesis. The 3′-proximal end of the genome encodes five structural proteins, including hemagglutinin-esterase (HE), spike (S), small membrane/envelope (E), integral membrane (M), and nucleocapsid (N), as well as five ORFs encoding the accessory proteins ns2, ns4.9, ns4.8, ns12.7, and protein I, whose ORF is located entirely within the N gene [5,7]. 

Similar to other RNA viruses, coronaviruses can adapt rapidly to changing ecological niches due to high mutation rates and recombination frequencies [8,9]. This plasticity may help BCoV overcome host barriers to infect other species and genetically adapt to a new host environment. For example, BCoV-like viruses have been detected in many species of wild and domestic ruminants as well as camelids [10,11,12,13,14,15,16,17]. Furthermore, BCoV cross-species transmissions have led to the establishment of separate virus lineages in humans (human coronavirus OC43), pigs (porcine hemagglutinating encephalomyelitis virus), horses (equine coronavirus), and dogs (canine respiratory coronavirus) (reviewed in [18]). Given the high genetic and antigenic relatedness of this group of viruses, they have been grouped into a single virus species named *Betacoronavirus 1* (b1CoV). These viruses display diverse genome structures in the accessory genes encoding ns4.9 and ns4.8. While the functions of these accessory proteins are unknown, they have been hypothesized to be niche-specific and play a role in virus tropism [19,20,21]. The hemagglutinin-esterase (HE) protein may also increase the host range of BCoV by expanding the number and/or types of cells the virus can infect [22].

The presence of HE is unique to the *Embecovirus* lineage of betacoronaviruses and was presumably acquired in a recombination event with influenza C virus [23]. Both HE and S proteins are found on the surface of the viral particle and are involved in binding to host cells through engagement of the 9-*O*-Acetylated-Sialic acid (9-*O*-Ac-Sia) on the cell surface. HE also possesses an esterase domain, which has receptor-destroying enzymatic activity capable of removing 9-*O*-Ac-Sia from the surface of cells [24]. Cleavage of the receptor is important for virus release as it allows newly formed virions to disseminate from the cell surface. Thus, HE and S are functionally interdependent and have co-evolved to balance attachment and release from host cells [25]. The S protein alone, however, mediates cell infection, suggesting that receptor destruction is the major function of HE [26,27]. 

Adaptive mutations in HE also contribute to shifts in host and tissue tropism and contribute to host selectivity following zoonotic events [28,29]. For example, human coronavirus OC43 HE lost receptor binding function in a progressive accumulation of mutations as the virus adapted to replication in human airways [28]. This was also accompanied by a change in HE-mediated receptor destruction. The most dramatic change in HE occurred in the murine betacoronaviruses where receptor specificity changed from 9-*O*-Ac-Sia to 4-*O*-Ac-Sia through modest changes in the architecture of the receptor binding site [29,30]. Thus, the evolution of HE reflects viral adaptation to novel hosts with altered host specificity and tropism. 

The HE gene in BCoV has evolved by both the accumulation of amino acid changes and recombination. In 2019, a novel BCoV strain with a recombinant HE gene was first detected in dairy cattle in China [31]. In 2020, Abi et al. [32] further described the emergence of a novel BCoV variant in China with a 4-amino acid insertion in the receptor-binding domain (RBD) of the recombinant HE gene. This variant was first detected in samples collected in 2018 from dairy cattle with diarrhea. The biological consequence of this insertion remains unknown; however, this variant may have altered receptor binding, host range, and/or tissue tropism [32]. Despite being an economically important pathogen with zoonotic potential, the global genetic and antigenic diversity of BCoV is poorly characterized. Thus, the global distribution of this variant is unknown. Our laboratories have had an interest in BCoV for some time and had been generating complete genome sequences directly from clinical samples collected from U.S. cattle with respiratory or enteric disease. Here, we report the genetic characterization of 78 genome sequences with complete coding sequence, including the identification of 11 isolates with a 4-amino acid insertion in HE similar to that first reported in China, and a single isolate with a 4-amino acid deletion in the HE RBD. Biological characterization revealed that these variants maintain esterase activity and binding to 9-*O*-Ac-Sia.

## 2. Materials and Methods

### 2.1. Samples and Ethics Statement

Respiratory and enteric samples used in this study came from the US Meat Animal Research Center (USMARC) cattle population (Clay Center, NE, USA) or samples submitted for diagnostic testing to veterinary diagnostic laboratories at the University of Nebraska-Lincoln, Nebraska Veterinary Diagnostic Center (NVDC) or South Dakota State University (SDSU). The USMARC cow-calf herd includes approximately 7000 cows managed in smaller herds across 34,000 acres of land. Until 2020, the USMARC cattle were managed as a closed herd. Nasal and fecal samples collected at the USMARC were done under the approval of multiple projects to study BCoV infection dynamics and immunity (Institutional Animal Care and Use Committee approval numbers: 24, 63, 74, and 97). The procedures for handling cattle complied with the Guide for the Care and Use of Agricultural Animals in Agricultural Research and Teaching (FASS, 2010). Samples were stored at −80 °C until prepared for sequencing. Diagnostic samples were anonymized for reporting. A total of 27 enteric samples (25 fecal, 2 intestinal) and 51 respiratory samples (49 nasal swabs, 2 lungs) were collected from cattle in 12 states in the U.S., though most of the samples (61/78) were from Nebraska (Table S1). Calves at USMARC did not receive a BCoV-containing vaccine, however, samples from the diagnostic laboratories came from cattle with unknown vaccination histories. 

### 2.2. Sample Preparation and Illumina Sequencing

Real-time reverse transcription polymerase chain reaction (RT-qPCR) was used to identify BCoV-positive clinical samples. Samples from USMARC were screened using a previously described primer and probe set [33] alone or in a multiplexed assay [34]. Samples from the diagnostic laboratories were screened according to their protocols. For samples from NVDC, RT-qPCR testing utilized the same primer and probe sequences as above in a multiplexed assay. Samples with an RT-qPCR cycle threshold (Ct) of less than 30 were prepared for sequencing. For the 61 samples sequenced at USMARC (identified by isolate names beginning with MARC/ or VDC/) the following methods were used: Fecal samples (n = 27) were diluted approximately 1:10 with sterile phosphate buffered saline (PBS), vortexed, and centrifuged at 10,000× *g* for 2 min at 4 °C. Nasal swabs (n = 34) were collected into minimal essential medium or viral transport medium and similarly clarified by centrifugation at 10,000× *g* for 2 min at 4 °C. Approximately 100 mg of intestinal tissue (n = 2) was homogenized in 1mL PBS using a polytron homogenizer. PBS was clarified by centrifugation at 18,000× *g* for 3 min at 4 °C. Clarified samples (250 uL) were treated with 20 U RNase One (Promega, Madison, WI, USA) and 30 U Turbo DNase (Ambion, Austin, TX, USA) in 1× DNase buffer (Ambion) at 37 °C for 90 min to degrade unprotected host and environmental nucleic acids. To ensure continuous DNase activity, 10 U of DNase was added to the sample every 30 min during the 90 min incubation. The remaining nucleic acids were isolated using Trizol LS (Invitrogen, Waltham, MA, USA) according to the manufacturer’s specifications. A final DNase treatment was performed to remove final traces of DNA from the RNA preparation. 

RNA libraries were prepared as previously described [35]. Briefly, 100 nanograms of total RNA were used as input material for the Illumina TruSeq RNA Sample Preparation Kit (Illumina, San Diego, CA, USA). Libraries were prepared as specified by the manufacturer’s protocol without the initial step of poly-A selection. Libraries were sequenced at the USMARC core facility on the Illumina MiSeq or NextSeq instrument. Raw sequence reads were processed using Geneious Prime software (v2021.1.1; Biomatters, Auckland, New Zealand). Index adapters and low-quality reads were removed using BBDuk (v38.37) as implemented in Geneious. Assembly of the viral genomic sequence was accomplished using template-assisted assembly, where trimmed reads were mapped to a reference genome (Mebus GenBank accession number U00735). The consensus genomes were manually inspected and annotated according to the NC_003045 BCoV-ENT genome and annotations in the ViPR database. GenBank accession numbers for the assembled genomes are listed in Table S1.

For the 17 genomes sequenced at SDSU, (identified by isolate names beginning with SDSU/), the following methods were used. In brief, samples were clarified by centrifugation and digested with a cocktail of nucleases [36]. Nucleic acids were isolated using the QIAamp Viral RNA Minikit (Qiagen, Hilden, Germany). Reverse transcription was then performed using barcoded random hexamers using the SuperScript III first-strand synthesis system (Invitrogen), followed by second-strand synthesis using Sequenase version 2.0 DNA polymerase (ThermoFisher Scientific, Waltham, MA, USA). DNA was purified using a Monarch PCR & DNA cleanup kit (New England Biolabs, Ipswich, MA, USA) followed by amplification by PCR using barcode primers. Amplified DNA was purified using the Monarch PCR & DNA cleanup kit and quantified using a Qubit 4 fluorometer (Invitrogen). Sequencing libraries were prepared using the Nextera XT library preparation kit (Illumina) and sequenced on a MiSeq using paired 151 bp reads. Reads were assembled *de novo* using CLC Genomics version 20. Contigs were identified by BLASTx as implemented by OmicsBox version 2.0.36. When complete coding genomes were not assembled by *de novo* methods, the most similar complete BCoV genome identified by BLASTx was used as a reference for guided assembly. Genomes were annotated as described above. 

### 2.3. Phylogenetic Analysis

To augment the newly reported genome sequences, full-length BCoV genome sequences were downloaded from the GenBank database in June of 2022 (Table S2). Sequences were aligned using multiple methods to find the alignment giving the most strongly supported neighbor net phylogenetic network in SplitsTree6 v 6.0 [37]. SplitsTree was chosen for phylogenetic inference as it can accommodate recombinant genomes in the dataset [37,38]. The complete methods are detailed on this internet site for protocol sharing (link: dx.doi.org/10.17504/protocols.io.kqdg3pyeql25/v3). Whole genome sequences, HE genes, and spike genes were aligned using MAFFT v7.450 with globalpair accuracy methods using the PAM (JTT) 100 substitution matrix [39,40]. Due to variable lengths in the 5′ and 3′ untranslated regions (UTRs), these sequences were trimmed from whole genome sequence alignments for downstream analyses. HE and spike gene alignments were deduplicated so only unique sequences were included in the downstream analyses. If a unique gene sequence was shared by more than one BCoV isolate, the sequence was designated with a sequence identifier comprised of a concatenation of isolate names separated by a colon or a double underscore, depending on downstream program allowances. Alignments were analyzed in SplitsTree6 using the Hamming distances method (default settings) and the Neighbor Net method [41] to obtain a Splits Network visualization [42]. Networks were constructed using the “CarefulMethod” inference algorithm. A maximum likelihood tree was also built from the whole genome sequence alignment using RaxML v.8.2.12. Statistical support for the tree was tested using 2000 bootstrap analyses followed by a maximum likelihood analysis. The final best tree was visualized using interactive tree of life (iToL) version 6.5.8.

### 2.4. Recombination Detection in HE Gene Sequences

For recombination detection in the HE gene, two datasets were used. The first included all *Betacoronavirus* HE gene sequences in the NCBI nucleotide database (September 2022) with 98% or greater coverage of the 1275 nt HE consensus sequence. To obtain these, Geneious Prime was used to extract the consensus HE sequence from the alignment of unique HE gene sequences from the 192 BCoV genomes analyzed in this study. A discontinuous megablast search (E-expectation value 1 × 10^−20^) of the NCBI nt database against the 1275 bp consensus BCoV HE gene consensus sequence retrieved 824 *Betacoronavirus* sequences, including those isolated from bovine and non-bovine hosts. Of these sequences, 714 had approximately 98.4% or more coverage of the query BCoV consensus sequence, thus having a minimum length of 1255 nt. Below this threshold, query coverage dramatically dropped. HE gene sequences were deduplicated leaving 492 unique HE gene sequences. If a unique HE gene sequence was shared by more than one coronavirus isolate, the sequence was designated with a sequence identifier comprised of a concatenation of GenBank accession numbers separated by a double underscore (Table S3). The 492 unique HE genes were aligned using MAFFT with the globalpair methods using the PAM (JTT) 100 substitution matrix. Recombination detection was performed using RDP5 [43], which used RDP [44], GENECONV [45], Bootscan [46], Maxchi [47], Chimaera [48], SiSscan [49] and 3Seq [50] as methods for recombination detection. A full exploratory scan was run, and recombination events were individually inspected. The second dataset included only those *Betacoronavirus* HE sequences with ‘bovine coronavirus’ included in the sequence description. From the 714 *Betacoronavirus* sequences, we filtered them to 345 BCoV HE sequences. These were deduplicated, resulting in 256 unique HE gene sequences (Table S4). These HE gene sequences were aligned using MAFFT with the globalpair methods using the PAM (JTT) 100 substitution matrix and analyzed in RDP5. Alignments were also analyzed in SplitsTree6 as described above. 

### 2.5. Expression of Wild Type and Variant Hemagglutinin Esterases

The complete HE gene from BCoV 18-25432 was synthesized with *BamHI* and *PacI* sites immediately adjacent to the start and stop codons, respectively, and cloned into the *BamHI* and *PacI* sites of pFAST-Bac. Strain 18-25432 was previously isolated from a bovine nasal swab from a clinical disease diagnostic submission and was used for serum neutralization assays in our SDSU laboratory. Likewise, the same HE gene was synthesized with a 12 nt insert encoding amino acids ‘KATV’ at positions 212–215 or a 12 nt deletion of nucleotides encoding ‘NGKF’ at positions 208–211. Plasmids were co-transfected into Sf9 cells along with linear baculovirus DNA (AB Vector, San Diego, CA, USA) using Profectin as per the manufacturer’s instructions. On day 5 post-transfection, 0.5mL of transfection culture supernatants were used to inoculate 250 mL shake flasks containing 50 mL of Sf9 cells at 1 × 10^6^ cells/mL in the Sf-900 II SFM medium (Gibco, Waltham, MA, USA). HE is a membrane protein that is expected to be present on the surface of the Sf9 cell membrane as well as the baculovirus membranes. Thus, crude cultures containing Sf9 cells and baculovirus were harvested by transfer to 4 °C on day 4 when cell viability was < 70%. Esterase activity was determined using 4-nitrophenyl acetate (NPA). Cultures were diluted 1:4 before being combined with an equal volume of 0.7 mM NPA in PBS. Absorbance was measured at 405 nm. Esterase activity was measured at 30 min and analyzed by one-way ANOVA and Tukey’s multiple comparison tests.

### 2.6. Hemagglutinin Esterase Lectin Activity

HE lectin (HEL) activity was determined in a solid phase binding assay. One hundred microliters of a 0.1 mg/mL solution of bovine submaxillary mucin (BSM) in PBS were used to coat Immulon 2 HB 96-well plates overnight at 4 °C followed by blocking with PBS+1% bovine serum albumin. Serial 2-fold dilutions of virus in PBS were incubated on the BSM plates for 1 h at 4 °C followed by three washes with PBS+0.05% tween 20 (PBST). Next, 100 µL of 100 µM 4-Methylumbelliferyl acetate was added to the wells and incubated at 37 °C for 1 h before the addition of 100% ethanol. Fluorescence was measured with excitation at 365 nm and emission at 450 nm. Lectin activities were analyzed by one-way ANOVA and Tukey’s multiple comparison tests. 

### 2.7. Hemagglutinin Esterase Structure Modeling

AlphaFold v2.2.2 [51] was downloaded from the GitHub repository and installed according to directions, as were the full databases including those supporting the AlphaFold-Multimer model. Two identical HE protein sequences were folded as a complex for each of the HE gene length variants: HE420 (deletion variant), HE424 (wild type), and HE428 (insertion variant) protein sequences. Briefly, for each variant, a single FASTA file was constructed with two copies of the variant sequence, with either an _A or _B suffix used in the sequence identifier to distinguish the two copies. For example, the HE420 variant FASTA file was called HE420_dimer.faa with the run_docker.py script executed as described in the AlphaFold GitHub README. In the case of the HE420 variant, the run_docker.py script was called using --fasta_paths=<HE420_dimer.faa>, --max_template_date=2022-01-1, --model_preset=multimer, --data_dir= <alphafold data directory> where text with < > includes the full path to the file or directory. For each variant, the PDB structure named ranked_0.pdb, the structure with the highest confidence, was selected to be the best representation of the biologically relevant structure. The three HE pdb files were loaded into a molecular graphics system (PyMOL Molecular Graphics System v2.5.3; Schrödinger, LLC, New York, NY, USA), aligned, and rotated to view the variant region. 

## 3. Results

### 3.1. Whole Genome Sequence Analysis 

Seventy-eight complete or near complete genomes were sequenced and assembled directly from enteric or respiratory samples collected between 2013 and 2022 from 27 enteric disease cases and 51 respiratory disease cases from NE (n = 61), WY (n = 1), KS (n = 1), ND (n = 1), OR (n = 1), PA (n = 1), SD (n = 2), CA (n = 3), TX (n = 3), CO (n = 1), WI (n = 1), and MN (n = 2) (Table S1). Genomes ranged from 30,682 to 31,039 nucleotides (nt) in length with mean coverage between 16 and 125,563-fold. 

**Figure 1:**
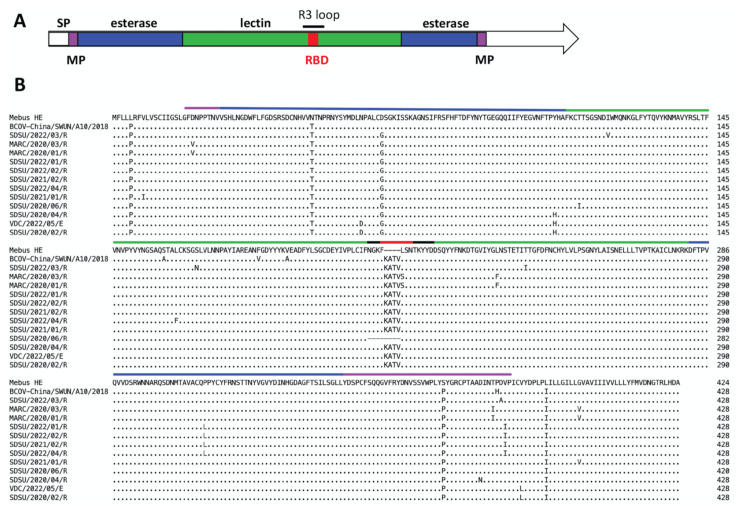
Alignment of variant HE proteins with HE from the reference BCoV Mebus genome. Panel A. HE protein map with domains annotated according to [28] with the signal peptide (SP) colored white, the lectin domain colored green, the esterase domain colored blue, and the membrane-proximal (MP) domain colored pink. The R3 loop and the receptor binding domain (RBD) are also annotated. Panel B. Alignment of 12 HE variant proteins from this study with the HE-insertion variant from China (GenBank accession no. MN982199, isolate BCoV-China/SWUN/A10/2018) and the reference Mebus sequence. Amino acids divergent from the reference Mebus sequence are shown while homologous amino acids are represented as a dot.

Comparative analyses of the 78 BCoV genomes revealed variable lengths in seven of the ORFs (Table S1). ORFs encoding the nonstructural proteins ns4.9 and ns4.8 had the highest variability between genomes. The length of ns4.9 ranged from 120 nt (n = 1) to 36 nt (n = 5); with most of the genomes containing a 90 nt ORF (n = 70). All of these differ from the reference Mebus genome which contains a 132 nt ORF. The length of ns4.8 ranged from 24 nt (n = 12) to 129 nt (n = 7), with most of the genomes containing a 126 nt ORF (n = 56). This also differed from the reference Mebus genome which contains a 138 nt ORF. Eleven genomes were found to have an insertion of 12 nt (AAGGC[U/C]ACUGUU) in the R3 loop of the HE gene resulting in the insertion of the amino acids ‘KATV’ after F_211_ (Figure 1). The R3 loop is one of five surface-exposed loops and is known to be essential for receptor binding. These 11 sequences came from nasal swab samples (n = 10) or feces (n = 1) collected between 2020 and 2022 from the states of NE, CO, CA, and WI (Table S1). One additional genome sequenced from a respiratory sample collected in Nebraska had a deletion of 12 nt in the R3 loop of the HE gene resulting in the deletion of the amino acids _208_NGKF_211_. None of the ORF sequence variants correlated with disease type; however, 11/12 of the HE variants were from respiratory disease samples. 

### 3.2. Phylogenetic Analysis

**Figure 2:**
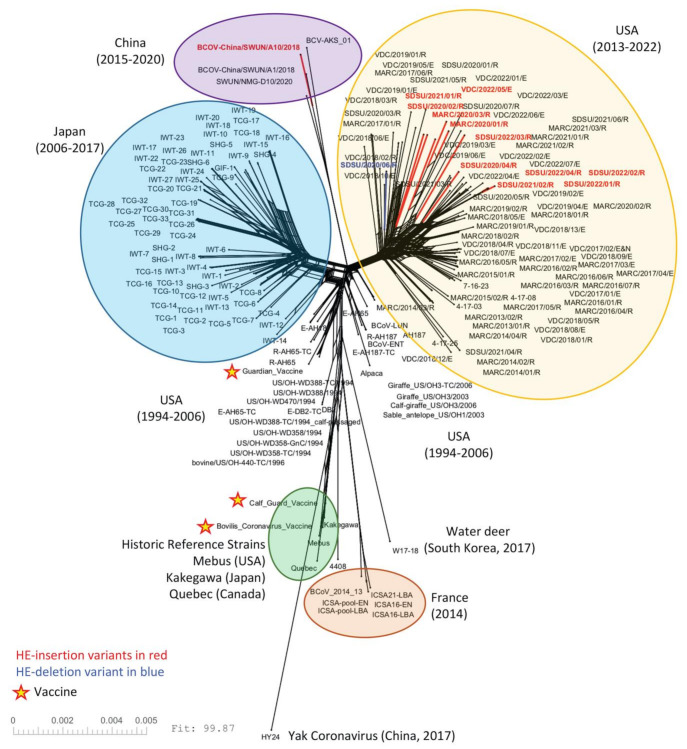
SplitsTree NeighborNet phylogenetic network of 192 BCoV genomes. The complete coding region of 192 genomes was aligned using MAFFT and analyzed with SplitsTree6. A list of the genomes used in this analysis can be found in Table S2. HE-insertion variants are in red and the single HE-deletion variant is in blue. The three vaccine strains are marked with a star.

**Figure 3:**
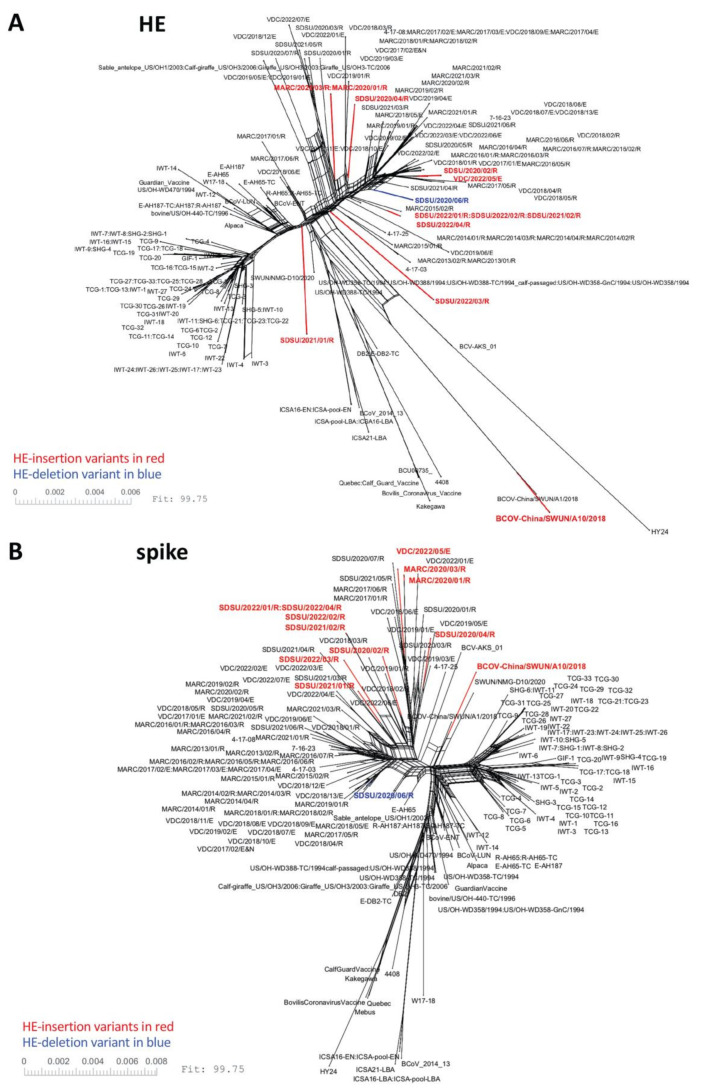
SplitsTree NeighborNet phylogenetic networks of 192 BCoV HE and spike genes. One hundred and ninety-two HE genes (**A**) or spike genes (**B**) were deduplicated, aligned using MAFFT, and analyzed with SplitsTree6. A list of the genomes used in this analysis can be found in Table S2.

A total of 192 BCoV genome sequences were used for phylogenetic analyses, including 78 genomes from clinical samples as well as three vaccine strains that were sequenced for this study. BCoV isolates could not be phylogenetically differentiated by clinical source (respiratory or enteric) when analyzed at the complete genome level (Figure 2 and Figure S1) or when looking at individual ORFs (Figure 3). Rather, respiratory and enteric isolates tended to cluster together by geographical origin and date of isolation rather than by disease presentation. 

The 11 U.S. genomes containing the 12 nt HE-insertion did not cluster together phylogenetically with the HE-insertion variant from China (GenBank accession no. MN982199, isolate BCoV-China/SWUN/A10/2018) when analyzed by complete genomes (Figure 2) or by the HE or spike gene sequences (Figure 3). Similarly, evaluation of the whole genome RaXML maximum likelihood tree similarly suggests that the U.S. and Chinese HE variants are evolving on distinct branches of the tree, with a bootstrap value of 88 for the branch linking all U.S. HE-insertion variants and 100 for the grouping of Chinese genomes with the Chinese HE variant. The bootstrap support for linking the Chinese branch to the U.S. branch falls to 55, a value that does not support collapsing the U.S. and Chinese variants into a single monophyletic group, especially given the much higher bootstrap support for the individual Chinese and U.S. variant groupings (Figure S1). These results imply that the U.S. and Chinese genomes possessing the 12 nt insertion do not share a common most recent ancestor. The phylogenetic analysis of only the HE gene similarly shows that the U.S. and Chinese HE-insertion variants do not share a common most recent ancestor. 

### 3.3. Recombination Detection

**Figure 4:**
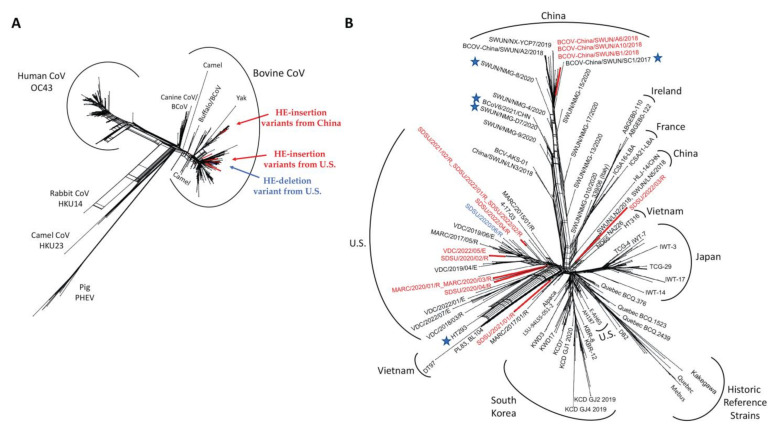
SplitsTree NeighborNet phylogenetic networks of HE gene sequences used in recombination detection. (**A**) NeighborNet of 491 unique *Betacoronavirus* HE gene sequences. GenBank accession OK391229 was removed from phylogenetic analysis due to poor sequence quality. (**B**) NeighborNet of 256 unique BCoV HE gene sequences. Stars represent the HE gene sequences flagged as potential recombinant sequences by RDP5. HE-insertion variants are colored red and the single HE-deletion variant is blue.

To determine whether the 12 nt HE insertions were acquired by RNA recombination, 492 unique *Betacoronavirus* HE gene sequences available in the NCBI nt database, including those from bovine and non-bovine hosts, were analyzed (Figure 4A). A total of eight recombination events were detected in 25 unique HE sequences with various levels of support (Table S5). A strong recombination signal was detected in dromedary camel coronavirus HE gene sequences, which were previously reported to have multiple cross-species recombination events with related Betacoronaviruses [52]. A weak recombination signal was detected in five BCoV HE sequences (Accession no. ON142319 (BCoV6/2021/CHN), ON142316 (BCoV3/2021/CHN), MW711311 (SWUN/NMG-D7/2020), M80842 (BRCV-G95), and MK045995 (HT293)). Parental sequences and statistical support for each recombination event are in Table S5. Given that there was no significant evidence of cross-species recombination events identified in the BCoV HE genes, recombination analyses were repeated with a smaller dataset containing 256 unique BCoV HE genes (Figure 4B). Four unique recombination events were detected in five HE gene sequences with various levels of support (Table S6). ON142319 (BCoV6/2021/CHN) had the highest support, with the recombination event detected by four methods in RDP5. MW711311 (SWUN/NMG-D7/2020), MK1095135 (BCOV-China/SWUN/SC1/2017), MW711317 (SWUN/NMG-8/2020), and MK045995 (HT293) were identified as recombinant by two methods (Table S6). No U.S. isolates sequenced in this study were identified as possessing a recombinant HE gene, including the HE variants, nor were the 12 nt HE-insertion variants from China. 

### 3.4. Baculovirus-Mediated Expression of Hemagglutinin Esterase Variants in Sf9 Cells

**Figure 5:**
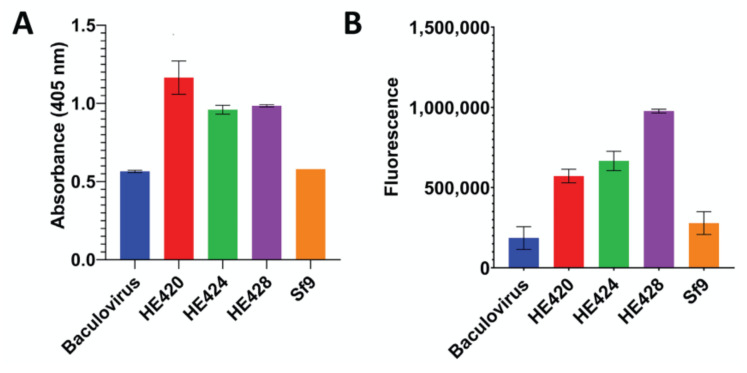
Functional Characterization of HE gene length variants. The full-length (HE424), insertional variant (HE428), or deletion variant (HE420) hemagglutinin-esterase genes were expressed in baculovirus. Esterase activity (panel **A**) and ability to bind 9-*O*-Ac-Sia (panel **B**) were measured with uninfected cells (Sf9) and cells infected with a baculovirus encoding an unrelated gene (Baculovirus) included as negative controls. A. The esterase activity between different HE variants was measured at 30 min after the start of the reaction. Error bars represent the standard deviation. The data were analyzed using a one-way ANOVA that showed significant variations (*p* = 0.0002). Tukey’s multiple comparison analyses showed that esterase activity of all HE variants were significantly higher than both Baculovirus and Sf9 negative controls (*p* < 0.05). B. The ability to bind the 9-*O*-Ac-Sia was assayed on bovine submaxillary mucin. Binding to ligand was significantly greater for all HE variants than Sf9 and Baculovirus controls *(p* < 0.05).

The complete HE gene from isolate 18-25432 (HE424) was synthesized and cloned into the baculovirus expression vector pFAST-Bac, along with isogenic alleles containing the 12 nucleotide insertion (HE428) or deletion (HE420) identified in circulating viruses. All three baculovirus HE expression constructs had esterase activity greater than baculovirus and Sf9 control cultures (Figure 5A), suggesting that the identified mutations in the HE receptor binding domain did not appreciably affect enzymatic activity. However, due to the lack of an anti-HE antibody, we were unable to normalize HE expression levels between baculovirus preparations. The ability of the HE mutants to bind the receptor 9-*O*-Ac-Sia was assessed using BSM. All three baculovirus HE protein variants bound BSM coated plates at levels greater than baculovirus and Sf9 control cultures, suggesting that the identified mutations did not abolish the ability of HE to bind 9-*O*-Ac-Sia (Figure 5B). In summary, these HE gene variants appear to maintain esterase activity and binding to 9-*O*-Ac-Sia.

### 3.5. Hemagglutinin Esterase Structural Modeling

**Figure 6:**
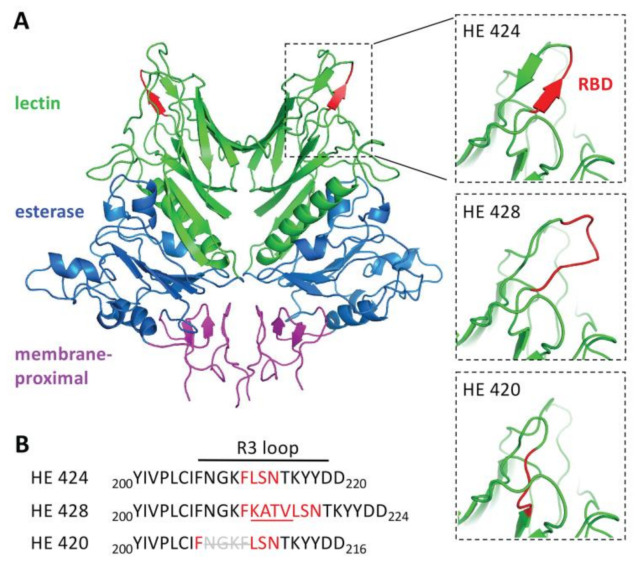
Structural modeling of HE gene length variants. Structural models of the homodimeric hemagglutinin-esterase protein (HE424) and mutants containing a 4-amino acid insertion (HE428) or deletion (HE420) in the receptor binding domain. A. Domains of HE424 were colored according to [28] with the lectin domain colored green, the esterase domain colored blue, and the membrane-proximal domain colored pink. The smaller panels zoom in on the R3 loop with the residues in the receptor binding domain (RBD) colored red. B. Sequences of the HE protein variants in the region containing the R3 loop.

To determine how the insertion or deletion in the HE gene sequence might affect protein structures, the amino acid sequences for HE420, HE424 and HE428 were modeled. Superimposition of the predicted HE proteins found structural changes limited to the R3 loop containing the receptor binding domain (RBD). Both the insertion (HE 428) and deletion (HE 420) variants disrupted the beta-sheet secondary structure that formed the RBD (Figure 6). 

## 4. Discussion

In this study, we report 78 complete or near-complete BCoV genome sequences, with 12 genomes containing HE gene length variants. The HE protein has a bimodular structure with a carbohydrate-binding lectin domain and an enzymatically active esterase domain [23,53,54] (Figure 1). The lectin domain mediates virion attachment to the receptor whereas the esterase domain results in receptor destruction and release of viral progeny. Residues F_211_, L_212_, S_213_ and N_214_ in the lectin domain are essential for receptor binding [23]. Eleven genomes sequenced in this study contained an insertion of 12 nt (AAGGC[U/C]ACUGUU) in the lectin domain, resulting in the insertion of the amino acids ‘KATV^’^ between F_211_ and F_212_ in the receptor binding domain (RBD, Figure 1) [23,32]. The nucleotide insertion from nine of these isolates was identical to the sequence recently reported by Abi et al., 2020 (AAGGCUACUGUU) [32] and the two isolates collected from USMARC had a synonymous nucleotide change that did not impact the amino acid sequence of the insertion (AAGGC**C**ACUGUU). These two USMARC isolates also had a leucine to serine substitution immediately following the insertion (L_212_ to S_212_; Figure 1). Ten of the genomes with the HE-insertion variant from this study were collected beginning in 2020 from non-epidemiologically linked cases in Nebraska, Colorado, California, and Wisconsin, and are the first report of this variant outside of China. While absent in the 48 samples collected prior to 2020, 11 out of 30 (37%) samples collected since 2020 were positive for the HE-insertion variant, suggesting that this variant is emerging in cattle. Interestingly, 10 of 11 HE-insertion variants were identified in samples from cattle with respiratory disease. However, given that the field samples used in this study were non-randomly sourced from a limited geography, the prevalence and ecology of the HE variant isolates in the U.S. remains uncertain. 

The origin of HE variants in the U.S. and China remains unknown. Phylogenetic analysis of complete genomes and HE gene sequences revealed that the HE-insertion variants from the U.S. are in a group distinct from those containing the Chinese HE variants. Yet, it is still possible that the HE-insertion variants acquired this 12 nt insertion by recombination with an ancestral virus with the 12 nt insertion. Intriguingly, one U.S. HE-insertion variant, SDSU/2022/03R, clustered near the previously reported non-recombinant HE sequences from China (Figure 4B) [32]. However, no recombination signal was detected the HE-insertion variants from the U.S. and China using RDP5. This contrasts with Abi et al., 2020, that reported the HE-insertion variants from China contained a recombinant HE gene [32]. However, this recombination event was observed in HE genes with and without the 12 nt insertion, suggesting the 12 nt insertion occurred independent of, and after, the recombination event that was detected. The recombination block in the Chinese isolates contained two common amino acid variants: F181V and P,S158A [32]. Neither of these amino acid variants were observed in any of the U.S. HE-insertion variants (Figure 1). Thus, there is no evidence to support the hypothesis that one of the U.S. or Chinese isolates identified to date serves as the ancestral isolate for the 12 nt insertion. We acknowledge that there may be one or more underlying recombination events that give rise the phylogeny observed, however, the high homology of BCoV HE gene sequences means that recombination signals can be difficult to confidently identify. Based on available evidence, the most parsimonious explanation for to the 12 nt insertion occurring in two different BCoV lineages is convergent evolution. However, we posit that because of (1) the rapid evolution of BCoV and (2) the paucity of complete BCoV genomes from across the globe, it is possible that the putative common ancestor of both the U.S. and Chinese HE-variants is not represented in public databases. Therefore, additional whole genome sequencing of historical isolates from underrepresented geographical regions will be required to understand the origin and spread of these variants.

In addition to the HE-insertion variants, a single genome assembled from a lung sample contained a novel 12 nt deletion in the HE receptor binding domain, resulting in the deletion of the amino acids _208_NGKF_211_ (Figure 1). The phenylalanine (F) at position 211 plays an essential role in forming a hydrophobic pocket that engages the receptor (the 5-N-acetyl group of 9-*O*-Ac-Sia) and mutation of F_211_ to alanine abolished receptor binding [23]. Interestingly, deletion of _208_NGFK_211_ places F_207_ immediately adjacent to L_212_, S_213_ and N_214_ which bind to the receptor via hydrogen bonding. Thus, we hypothesized that the F_207_ in the HE deletion variant may be able to complement for deletion of F_211_ to allow receptor binding (Figure 1). However, given the structural changes observed in the RBD by protein modeling (Figure 6) it was unclear if this variant would still bind the receptor similar to the wild type HE protein. 

To determine the potential impact of these HE deletion/insertion variants on receptor binding and esterase activity, isogenic HE proteins containing either the insertion or deletion in the lectin domain were cloned into a baculovirus expression vector. Prior 3D modeling based on the crystal structure of BCoV HE [23] predicted that the four amino acid insertion would alter the spatial conformation of the R3 loop containing the receptor-binding site [32] which could potentially impact receptor binding. Binding assays using BSM demonstrated that HE proteins bearing the _208_NGKF_211_ deletion or the _212_KATV_215_ insertion were able to bind receptors similar to canonical (wild type) HE, suggesting that the mutations do not abolish receptor binding (Figure 5). However, because we lacked an anti-HE antibody to normalize HE expression levels between the different baculovirus-expressed HE preparations, we cannot exclude the possibility that there are more subtle differences in receptor binding and esterase activity. Differences in HE glycosylation by Sf9 cells, as compared to bovine cells, may also impact HE binding and enzymatic activity. Thus, whether observed or predicted changes in receptor binding and destruction may alter tissue tropism and/or host range remains unknown. 

HE and S glycoproteins both bind to the 9-*O*-Ac-Sia receptor; however, the S glycoprotein enables receptor binding and membrane fusion allowing virus entry, while the HE protein plays a critical role in viral egress from the cell, cleaving the receptor and allowing newly formed virions to disseminate from the cell surface. The opposing effects of S and HE have been tuned by evolutionary forces to maximize viral fitness [25]. A question that remains is whether certain spike genotypes allow viruses with changes in the HE receptor binding domain to remain viable. NeighborNet reconstruction revealed that while the HE genes with the insertional variant were observed in several clades throughout the tree (Figure 3), the spike genes from BCoV isolates containing the HE-insertion variant appeared to cluster together in fewer clades. Analysis of additional genomes is needed to determine whether there are certain motifs in spike that associate with HE insertional variants. Furthermore, given that both S and HE surface glycoproteins are targeted by neutralizing antibodies [55,56,57], it is also of interest to determine whether structural changes in the HE receptor binding domain alter antibody recognition of HE epitopes, and if these changes in antibody recognition could result in immune escape variants. Additional work is needed to address these important questions.

An additional early goal of this work was to compare respiratory and enteric isolates to determine whether there are genomic signatures related to respiratory or enteric tropism. Phylogenetically, the respiratory and enteric isolates clustered together by geographical origin and date of isolation rather than by disease presentation (Figure 2), which is consistent with previous findings [58,59,60,61,62]. To date, no distinct genetic or antigenic markers have been consistently identified in BCoVs associated with these distinct clinical presentations; however, few studies have analyzed complete genome sequences that came directly from clinical samples. In the present study, there were confounding factors (e.g., U.S. state of origin, year of isolation, etc.) and an insufficient sampling depth to make meaningful comparisons between respiratory and enteric isolates when epidemiologically linked samples were removed. Therefore, a more geographically diverse set of enteric and respiratory BCoV genomes adequately sampled across space and time is needed in the future for comparisons to identify potential tropism determinants. Nevertheless, it is possible that enteric and respiratory BCoVs are members of the same quasispecies and the outcome of infection is impacted by route of exposure, co-infections, or other host and environmental factors, individually or in combination, rather than specific genetic determinants of tropism [58,63].

## 5. Conclusions

The recent pandemic caused by SARS-CoV-2 has illustrated the importance of animal reservoirs of coronaviruses. Bovine coronavirus in particular has shown a propensity for interspecies transmission. Bovine coronavirus-like viruses have been detected in a large number of domestic and wild ruminants [64], and spillover events have led to the establishment of BCoV-like viruses in multiple species [21]. Apart from sporadic amino acid mutations, we did not observe any deletions or insertions in BCoV HE sequences prior to 2020. One concern is that the recent emergence of HE genes with insertions or deletions in the HE receptor binding domain may alter the host range of BCoV. A similar insertion into the R3 loop of the mouse hepatitis virus HE gene led to a change in receptor tropism to 4-*O*-Ac-Sia from 9-*O*-Ac-Sia [29]. We also noted length polymorphisms within genes encoding the 4.9 kDa and 4.8 kDa proteins, including large truncations in the predicted open reading frames. Deletions in this region of the genome for human CoV OC43 have been hypothesized to be associated with its adaptation to humans [65,66]. Therefore, further investigation of BCoV evolution and in particular these novel HE variants is warranted.

## Data Availability

The viral genome sequences generated during this study are available at NCBI with Accession numbers OP037365–OP037442. Alignments used for phylogenetic analysis can be found at Protocols.io. An interactive display of the maximum likelihood tree can be found at iTOL.

## Supplementary Materials

The following supporting information can be downloaded at: https://www.mdpi.com/article/10.3390/v14102125/s1, Figure S1: Maximum likelihood phylogenetic tree of 192 BCoV genomes. Table S1: Summary of 78 U.S. BCoV genomes characterized in this study; Table S2: BCoV genomes used for phylogenetic analysis. Table S3: Sequence IDs for 492 Unique *Betacoronavirus* HE gene sequences from NCBI. Table S4: Sequence IDs for 256 Unique BCoV HE gene sequences from NCBI. Table S5: Recombination detection in 492 unique *Betacoronavirus* HE gene sequences. Table S6: Recombination detection in 256 unique HE sequences with ‘bovine coronavirus’ in the description.

## References

[1] Hasoksuz M., Sreevatsan S., Cho K.O., Hoet A.E., Saif L.J. (2002). Molecular analysis of the S1 subunit of the spike glycoprotein of respiratory and enteric bovine coronavirus isolates. Virus Res..

[2] Saif L.J. (2010). Bovine respiratory coronavirus. Vet. Clin. N. Am. Food Anim. Pract..

[3] Griffin D., Chengappa M.M., Kuszak J., McVey D.S. (2010). Bacterial pathogens of the bovine respiratory disease complex. Vet. Clin. N. Am. Food Anim. Pract..

[4] Foster D.M., Smith G.W. (2009). Pathophysiology of diarrhea in calves. Vet. Clin. N. Am. Food Anim. Pract..

[5] Yoo D., Pei Y. (2001). Full-length genomic sequence of bovine coronavirus (31 kb). Completion of the open reading frame 1a/1b sequences. Adv. Exp. Med. Biol..

[6] Brierley I., Boursnell M.E., Binns M.M., Bilimoria B., Blok V.C., Brown T.D., Inglis S.C. (1987). An efficient ribosomal frame-shifting signal in the polymerase-encoding region of the coronavirus IBV. EMBO J..

[7] Senanayake S.D., Brian D.A. (1997). Bovine coronavirus I protein synthesis follows ribosomal scanning on the bicistronic N mRNA. Virus Res..

[8] Sanchez C.M., Gebauer F., Sune C., Mendez A., Dopazo J., Enjuanes L. (1992). Genetic evolution and tropism of transmissible gastroenteritis coronaviruses. Virology.

[9] Woo P.C., Lau S.K., Huang Y., Yuen K.Y. (2009). Coronavirus diversity, phylogeny and interspecies jumping. Exp. Biol. Med. (Maywood).

[10] Alekseev K.P., Vlasova A.N., Jung K., Hasoksuz M., Zhang X., Halpin R., Wang S., Ghedin E., Spiro D., Saif L.J. (2008). Bovine-like coronaviruses isolated from four species of captive wild ruminants are homologous to bovine coronaviruses, based on complete genomic sequences. J. Virol..

[11] Cebra C.K., Mattson D.E., Baker R.J., Sonn R.J., Dearing P.L. (2003). Potential pathogens in feces from unweaned llamas and alpacas with diarrhea. J. Am. Vet. Med. Assoc..

[12] He Q., Guo Z., Zhang B., Yue H., Tang C. (2019). First detection of bovine coronavirus in Yak (Bos grunniens) and a bovine coronavirus genome with a recombinant HE gene. J. Gen. Virol..

[13] Tsunemitsu H., el-Kanawati Z.R., Smith D.R., Reed H.H., Saif L.J. (1995). Isolation of coronaviruses antigenically indistinguishable from bovine coronavirus from wild ruminants with diarrhea. J. Clin. Microbiol..

[14] Hasoksuz M., Alekseev K., Vlasova A., Zhang X., Spiro D., Halpin R., Wang S., Ghedin E., Saif L.J. (2007). Biologic, antigenic, and full-length genomic characterization of a bovine-like coronavirus isolated from a giraffe. J. Virol..

[15] Majhdi F., Minocha H.C., Kapil S. (1997). Isolation and characterization of a coronavirus from elk calves with diarrhea. J. Clin. Microbiol..

[16] Wunschmann A., Frank R., Pomeroy K., Kapil S. (2002). Enteric coronavirus infection in a juvenile dromedary (Camelus dromedarius). J. Vet. Diagn. Investig..

[17] Chasey D., Reynolds D.J., Bridger J.C., Debney T.G., Scott A.C. (1984). Identification of coronaviruses in exotic species of Bovidae. Vet. Rec..

[18] Decaro N., Lorusso A. (2020). Novel human coronavirus (SARS-CoV-2): A lesson from animal coronaviruses. Vet. Microbiol..

[19] Mounir S., Talbot P.J. (1992). Sequence analysis of the membrane protein gene of human coronavirus OC43 and evidence for O-glycosylation. J. Gen. Virol..

[20] Gelinas A.M., Sasseville A.M., Dea S. (2001). Identification of specific variations within the HE, S1, and ORF4 genes of bovine coronaviruses associated with enteric and respiratory diseases in dairy cattle. Adv. Exp. Med. Biol..

[21] Vijgen L., Keyaerts E., Lemey P., Maes P., Van Reeth K., Nauwynck H., Pensaert M., Van Ranst M. (2006). Evolutionary history of the closely related group 2 coronaviruses: Porcine hemagglutinating encephalomyelitis virus, bovine coronavirus, and human coronavirus OC43. J. Virol..

[22] Saif L.J., Jung K. (2020). Comparative Pathogenesis of Bovine and Porcine Respiratory Coronaviruses in the Animal Host Species and SARS-CoV-2 in Humans. J. Clin. Microbiol..

[23] Zeng Q., Langereis M.A., van Vliet A.L., Huizinga E.G., de Groot R.J. (2008). Structure of coronavirus hemagglutinin-esterase offers insight into corona and influenza virus evolution. Proc. Natl. Acad. Sci. USA.

[24] Schultze B., Wahn K., Klenk H.D., Herrler G. (1991). Isolated HE-protein from hemagglutinating encephalomyelitis virus and bovine coronavirus has receptor-destroying and receptor-binding activity. Virology.

[25] Lang Y., Li W., Li Z., Koerhuis D., van den Burg A.C.S., Rozemuller E., Bosch B.J., van Kuppeveld F.J.M., Boons G.J., Huizinga E.G. (2020). Coronavirus hemagglutinin-esterase and spike proteins coevolve for functional balance and optimal virion avidity. Proc. Natl. Acad. Sci. USA.

[26] Popova R., Zhang X. (2002). The spike but not the hemagglutinin/esterase protein of bovine coronavirus is necessary and sufficient for viral infection. Virology.

[27] Storz J., Zhang X.M., Rott R. (1992). Comparison of hemagglutinating, receptor-destroying, and acetylesterase activities of avirulent and virulent bovine coronavirus strains. Arch. Virol..

[28] Bakkers M.J., Lang Y., Feitsma L.J., Hulswit R.J., de Poot S.A., van Vliet A.L., Margine I., de Groot-Mijnes J.D., van Kuppeveld F.J., Langereis M.A. (2017). Betacoronavirus Adaptation to Humans Involved Progressive Loss of Hemagglutinin-Esterase Lectin Activity. Cell Host Microbe.

[29] Langereis M.A., Zeng Q., Heesters B.A., Huizinga E.G., de Groot R.J. (2012). The murine coronavirus hemagglutinin-esterase receptor-binding site: A major shift in ligand specificity through modest changes in architecture. PLoS Pathog..

[30] Bakkers M.J., Zeng Q., Feitsma L.J., Hulswit R.J., Li Z., Westerbeke A., van Kuppeveld F.J., Boons G.J., Langereis M.A., Huizinga E.G. (2016). Coronavirus receptor switch explained from the stereochemistry of protein-carbohydrate interactions and a single mutation. Proc. Natl. Acad. Sci. USA.

[31] Keha A., Xue L., Yan S., Yue H., Tang C. (2019). Prevalence of a novel bovine coronavirus strain with a recombinant hemagglutinin/esterase gene in dairy calves in China. Transbound. Emerg. Dis..

[32] Abi K.M., Zhang Q., Zhang B., Zhou L., Yue H., Tang C. (2020). An emerging novel bovine coronavirus with a 4-amino-acid insertion in the receptor-binding domain of the hemagglutinin-esterase gene. Arch. Virol..

[33] Decaro N., Elia G., Campolo M., Desario C., Mari V., Radogna A., Colaianni M.L., Cirone F., Tempesta M., Buonavoglia C. (2008). Detection of bovine coronavirus using a TaqMan-based real-time RT-PCR assay. J. Virol. Methods.

[34] Workman A.M., Kuehn L.A., McDaneld T.G., Clawson M.L., Loy J.D. (2019). Longitudinal study of humoral immunity to bovine coronavirus, virus shedding, and treatment for bovine respiratory disease in pre-weaned beef calves. BMC Vet. Res..

[35] Workman A.M., Heaton M.P., Harhay G.P., Smith T.P., Grotelueschen D.M., Sjeklocha D., Brodersen B., Petersen J.L., Chitko-McKown C.G. (2016). Resolving Bovine viral diarrhea virus subtypes from persistently infected U.S. beef calves with complete genome sequence. J. Vet. Diagn. Investig. Off. Publ. Am. Assoc. Vet. Lab. Diagn. Inc.

[36] Hause B.M., Collin E.A., Anderson J., Hesse R.A., Anderson G. (2015). Bovine rhinitis viruses are common in U.S. cattle with bovine respiratory disease. PLoS ONE.

[37] Huson D.H. (1998). SplitsTree: Analyzing and visualizing evolutionary data. Bioinformatics.

[38] Huson D.H., Bryant D. (2006). Application of phylogenetic networks in evolutionary studies. Mol. Biol. Evol..

[39] Katoh K., Misawa K., Kuma K., Miyata T. (2002). MAFFT: A novel method for rapid multiple sequence alignment based on fast Fourier transform. Nucleic Acids Res..

[40] Katoh K., Standley D.M. (2013). MAFFT multiple sequence alignment software version 7: Improvements in performance and usability. Mol. Biol. Evol..

[41] Bryant D., Moulton V. (2004). Neighbor-net: An agglomerative method for the construction of phylogenetic networks. Mol. Biol. Evol..

[42] Dress A.W., Huson D.H. (2004). Constructing splits graphs. IEEE/ACM Trans. Comput. Biol. Bioinform..

[43] Martin D.P., Varsani A., Roumagnac P., Botha G., Maslamoney S., Schwab T., Kelz Z., Kumar V., Murrell B. (2021). RDP5: A computer program for analyzing recombination in, and removing signals of recombination from, nucleotide sequence datasets. Virus Evol..

[44] Martin D., Rybicki E. (2000). RDP: Detection of recombination amongst aligned sequences. Bioinformatics.

[45] Padidam M., Sawyer S., Fauquet C.M. (1999). Possible emergence of new geminiviruses by frequent recombination. Virology.

[46] Martin D.P., Posada D., Crandall K.A., Williamson C. (2005). A modified bootscan algorithm for automated identification of recombinant sequences and recombination breakpoints. AIDS Res. Hum. Retrovir..

[47] Smith J.M. (1992). Analyzing the mosaic structure of genes. J. Mol. Evol..

[48] Posada D., Crandall K.A. (2001). Evaluation of methods for detecting recombination from DNA sequences: Computer simulations. Proc. Natl. Acad. Sci. USA.

[49] Gibbs M.J., Armstrong J.S., Gibbs A.J. (2000). Sister-scanning: A Monte Carlo procedure for assessing signals in recombinant sequences. Bioinformatics.

[50] Lam H.M., Ratmann O., Boni M.F. (2018). Improved Algorithmic Complexity for the 3SEQ Recombination Detection Algorithm. Mol. Biol. Evol..

[51] Jumper J., Evans R., Pritzel A., Green T., Figurnov M., Ronneberger O., Tunyasuvunakool K., Bates R., Zidek A., Potapenko A. (2021). Highly accurate protein structure prediction with AlphaFold. Nature.

[52] So R.T.Y., Chu D.K.W., Miguel E., Perera R., Oladipo J.O., Fassi-Fihri O., Aylet G., Ko R.L.W., Zhou Z., Cheng M.S. (2019). Diversity of Dromedary Camel Coronavirus HKU23 in African Camels Revealed Multiple Recombination Events among Closely Related Betacoronaviruses of the Subgenus Embecovirus. J. Virol..

[53] Langereis M.A., Zeng Q., Gerwig G.J., Frey B., von Itzstein M., Kamerling J.P., de Groot R.J., Huizinga E.G. (2009). Structural basis for ligand and substrate recognition by torovirus hemagglutinin esterases. Proc. Natl. Acad. Sci. USA.

[54] Rosenthal P.B., Zhang X., Formanowski F., Fitz W., Wong C.H., Meier-Ewert H., Skehel J.J., Wiley D.C. (1998). Structure of the haemagglutinin-esterase-fusion glycoprotein of influenza C virus. Nature.

[55] Deregt D., Babiuk L.A. (1987). Monoclonal antibodies to bovine coronavirus: Characteristics and topographical mapping of neutralizing epitopes on the E2 and E3 glycoproteins. Virology.

[56] Storz J., Herrler G., Snodgrass D.R., Hussain K.A., Zhang X.M., Clark M.A., Rott R. (1991). Monoclonal antibodies differentiate between the haemagglutinating and the receptor-destroying activities of bovine coronavirus. J. Gen. Virol..

[57] Deregt D., Gifford G.A., Ijaz M.K., Watts T.C., Gilchrist J.E., Haines D.M., Babiuk L.A. (1989). Monoclonal antibodies to bovine coronavirus glycoproteins E2 and E3: Demonstration of in vivo virus-neutralizing activity. J. Gen. Virol..

[58] Suzuki T., Otake Y., Uchimoto S., Hasebe A., Goto Y. (2020). Genomic Characterization and Phylogenetic Classification of Bovine Coronaviruses Through Whole Genome Sequence Analysis. Viruses.

[59] Park S.J., Kim G.Y., Choy H.E., Hong Y.J., Saif L.J., Jeong J.H., Park S.I., Kim H.H., Kim S.K., Shin S.S. (2007). Dual enteric and respiratory tropisms of winter dysentery bovine coronavirus in calves. Arch. Virol..

[60] Bidokhti M.R., Traven M., Ohlson A., Baule C., Hakhverdyan M., Belak S., Liu L., Alenius S. (2012). Tracing the transmission of bovine coronavirus infections in cattle herds based on S gene diversity. Vet. J..

[61] Beuttemmuller E.A., Alfieri A.F., Headley S.A., Alfieri A.A. (2017). Brazilian strain of bovine respiratory coronavirus is derived from dual enteric and respiratory tropism. Genet. Mol. Res..

[62] Zhang X., Hasoksuz M., Spiro D., Halpin R., Wang S., Vlasova A., Janies D., Jones L.R., Ghedin E., Saif L.J. (2007). Quasispecies of bovine enteric and respiratory coronaviruses based on complete genome sequences and genetic changes after tissue culture adaptation. Virology.

[63] Ellis J. (2019). What is the evidence that bovine coronavirus is a biologically significant respiratory pathogen in cattle?. Can. Vet. J. La Rev. Vet. Can..

[64] Vlasova A.N., Saif L.J. (2021). Bovine Coronavirus and the Associated Diseases. Front. Vet. Sci..

[65] Vijgen L., Keyaerts E., Moes E., Thoelen I., Wollants E., Lemey P., Vandamme A.M., Van Ranst M. (2005). Complete genomic sequence of human coronavirus OC43: Molecular clock analysis suggests a relatively recent zoonotic coronavirus transmission event. J. Virol..

[66] Vijgen L., Lemey P., Keyaerts E., Van Ranst M. (2005). Genetic variability of human respiratory coronavirus OC43. J. Virol..

